# Inactivation of UDP-Glucose Sterol Glucosyltransferases Enhances *Arabidopsis* Resistance to *Botrytis cinerea*

**DOI:** 10.3389/fpls.2019.01162

**Published:** 2019-09-27

**Authors:** Nidia Castillo, Victoria Pastor, Ángel Chávez, Montserrat Arró, Albert Boronat, Victor Flors, Albert Ferrer, Teresa Altabella

**Affiliations:** ^1^Plant Metabolism and Metabolic Engineering Program, Centre for Research in Agricultural Genomics (CRAG), CSIC-IRTA-UAB-UB, Barcelona, Spain; ^2^Metabolic Integration and Cell Signalling Group, Plant Physiology Section, Department of Ciencias Agrarias y del Medio Natural, Universitat Jaume I, Castelló, Spain; ^3^Department of Biochemistry and Physiology, Faculty of Pharmacy and Food Sciences, University of Barcelona, Barcelona, Spain; ^4^Department of Biochemistry and Molecular Biomedicine, Faculty of Biology, University of Barcelona, Barcelona, Spain; ^5^Department of Biology, Healthcare and the Environment, Faculty of Pharmacy and Food Sciences, University of Barcelona, Barcelona, Spain

**Keywords:** *Arabidopsis*, biotic stress, *Botrytis cinerea*, camalexin, indole glucosinolates, JA signaling pathway, steryl glycosides

## Abstract

Free and glycosylated sterols are both structural components of the plasma membrane that regulate their biophysical properties and consequently different plasma membrane-associated processes such as plant adaptation to stress or signaling. Several reports relate changes in glycosylated sterols levels with the plant response to abiotic stress, but the information about the role of these compounds in the response to biotic stress is scarce. In this work, we have studied the response to the necrotrophic fungus *Botrytis cinerea* in an *Arabidopsis* mutant that is severely impaired in steryl glycosides biosynthesis due to the inactivation of the two sterol glucosyltransferases (UGT80A2 and UGT80B1) reported in this plant. This mutant exhibits enhanced resistance against *B. cinerea* when compared to wild-type plants, which correlates with increased levels of jasmonic acid (JA) and up-regulation of two marker genes (*PDF1.2* and *PR4*) of the ERF branch of the JA signaling pathway. Upon *B. cinerea* infection, the *ugt80A2;B1* double mutant also accumulates higher levels of camalexin, the major *Arabidopsis* phytoalexin, than wild-type plants. Camalexin accumulation correlates with enhanced transcript levels of several cytochrome P450 camalexin biosynthetic genes, as well as of their transcriptional regulators *WRKY33*, *ANAC042*, and *MYB51*, suggesting that the *Botrytis*-induced accumulation of camalexin is coordinately regulated at the transcriptional level. After fungus infection, the expression of genes involved in the indole glucosinolate biosynthesis is also up-regulated at a higher degree in the *ugt80A2;B1* mutant than in wild-type plants. Altogether, the results of this study show that glycosylated sterols play an important role in the regulation of *Arabidopsis* response to *B. cinerea* infection and suggest that this occurs through signaling pathways involving the canonical stress-hormone JA and the tryptophan-derived secondary metabolites camalexin and possibly also indole glucosinolates.

## Introduction

Steryl glycosides (SGs) are conjugated forms of sterols resulting from the attachment through a glycosidic bond of a sugar residue, most commonly a single glucose monomer, to the free hydroxyl group at C3 position of the sterol backbone (Ferrer et al., 2017). This reaction is catalyzed by UDP-glucose:sterol glycosyltransferase (SGT; E.C. 2.4.1.173), an enzyme that has been cloned and functionally characterized from different organisms (Grille et al., 2010) including several plant species (Warnecke et al., 1997; DeBolt et al., 2009; Chaturvedi et al., 2012; Li et al., 2014; Tiwari et al., 2014; Ramírez-Estrada et al., 2017). The hydroxyl group at C6 position of the sugar moiety can in turn be esterified with a long-chain fatty acid to form acyl steryl glycosides (ASG), although the enzyme responsible for this transformation has not been identified yet (Ferrer et al., 2017). The content of glycosylated sterols (SG + ASG) differs among plant species and tissues, but in general, these compounds are relatively minor components ranging from 10% to 30% of the total sterol fraction, although there are some exceptions in the Solanaceae family, as for instance tomato and potato, in which glycosylated sterols are the predominant form of sterols (Moreau et al., 2002; Furt et al., 2010; Nyström et al., 2012).

The role of free sterols (FSs) as key structural components of the plasma membrane has been known for a long time. Free sterols help to modulate the plasma membrane biophysical properties and hence its biological function and the activity of membrane-bound proteins (Carruthers and Melchior, 1986; Cooke and Burden, 1990; Grandmougin-Ferjani et al., 1997). Free sterols have also been recognized as important modulators of plant growth and development (Schrick et al., 2000; Schrick et al., 2002; He et al., 2003; Carland et al., 2010; Ovecka et al., 2010; Qian et al., 2013; Nakamoto et al., 2015), and glycosylated sterols are also emerging as important players in determining plasma membrane organization and functionality (Moreau et al., 2002; Grosjean et al., 2015; Cassim et al., 2019). Like FSs, glycosylated sterols are unevenly distributed in the plasma membrane, and it is currently accepted that SG and ASG are also highly enriched alongside with sterols, sphingolipids and selected proteins in liquid-ordered phase domains referred to as membrane rafts or DRM (sterol-enriched detergent-resistant membrane fraction). These dynamic assemblies of lipids and proteins appear to be involved in different plant cell processes including polarized cell growth, cell-to-cell communication, intracellular membrane trafficking, and signal transduction cascades enabling plants to respond to environmental changes (Mongrand et al., 2010; Zauber et al., 2014; Gronnier et al., 2018). However, the specific role of glycosylated sterols in regulating membrane properties and function still remains uncertain, although some experimental evidences support the view that a proper ratio of the glycosylated versus free forms of sterols in cell membranes is essential for normal plant cell function and overall plant performance. Thus, an *Arabidopsis* null mutant defective in the two SGTs present in this species, namely, UGT80A2 and UGT80B1 (DeBolt et al., 2009), displays highly reduced levels of glycosylated sterols in different plant organs that lead to multiple morphological and biochemical seed phenotypes (DeBolt et al., 2009), defects in the male gametophyte (Choi et al., 2014), and aberrant root epidermal cell patterning (Pook et al., 2017). Also, down-regulation of SGTs in agroinfiltrated *Withania somnifera* leaves leads to shortened plant height and leaf area compared to control plants (Singh et al., 2016).

Forward- and reverse-genetic approaches have also shown that changes in SGT expression levels are associated to altered responses of different plant species to abiotic stress conditions. An increased sensitivity to heat and cold stress has been reported in *Arabidopsis* and *W. somnifera* plants with reduced levels of SGT (Mishra et al., 2015; Singh et al., 2017), whereas enhanced tolerance to heat, cold, and salt stress has been associated to overexpression of SGT in *Arabidopsis*, tobacco, and *W. somnifera*, respectively (Mishra et al., 2013; Pandey et al., 2014; Saema et al., 2016). These observations are consistent with the induction of SGT genes in response to abiotic stress reported in tomato (Ramírez-Estrada et al., 2017), *W. somnifera* (Chaturvedi et al., 2012), and cotton (Li et al., 2014), and also with changes observed in the relative proportions of glycosylated sterols in the plasma membrane of oat, rye, and potato in association with cold acclimation and freezing tolerance (Palta et al., 1993; Takahashi et al., 2016), during tomato and apple fruit chilling and after tomato fruit rewarming (Whitaker, 1991; Whitaker, 1994; Rudell et al., 2011), in wheat leaves under high day and night temperature (Narayanan et al., 2016), and in *Arabidopsis* under drought stress conditions (Tarazona et al., 2015). On the contrary, the experimental evidence supporting a role for glycosylated sterols in mediating plant responses against biotic stress is far more limited. *Arabidopsis* and tobacco plants overexpressing *W. somnifera* SGT show increased resistance toward *Alternaria brassicicola* and *Spodoptera litura*, respectively (Pandey et al., 2014; Mishra et al., 2017), and basal immunity in *W. somnifera* plants is compromised after silencing of several members of the SGT gene family (Singh et al., 2016). However, it is still unclear whether these effects are due to the altered levels of glycosylated sterols or are actually triggered by the concomitant changes in the contents of other bioactive specialized plant defense compounds present in these species (Pandey et al., 2014; Singh et al., 2016; Mishra et al., 2017). The marked induction of specific members of the tomato and *W. somnifera* SGT gene families in response to methyl jasmonate (MeJA) further suggests a role for sterol glycosylation in plant response to biotic stress imposed by necrotrophic pathogens. However, the impact of this transcriptional response on the levels of steroidal glycoalkaloids in tomato and whitanolides in *W. somnifera* remains to be established. These defense compounds are not produced in the model plant *Arabidopsis thaliana*, which presents a rather scarce secondary metabolism. Consequently, the *Arabidopsis* double mutant *ugt80A2;B1* impaired in the SGs biosynthesis (DeBolt et al., 2009) is a very suitable tool to study the role of this kind of conjugated sterols in the plant defense response to pathogen attack, which involves changes at the transcriptional, biochemical, and physiological levels (AbuQamar et al., 2017).

When a pathogen is detected by the plant, it activates different layers of defense depending of the pathogen invasion stage. A first layer is constituted by a repertoire of plasma membrane pattern recognition receptors that perceive signals produced by the pathogen, known as pathogen- or microbe-associated molecular patterns (MAMPs), or plant-derived damage-associated molecular patterns (DAMPs) produced by the host upon pathogen infection (Bohm et al., 2014; Zipfel, 2014). This induces a basal disease resistance response called pattern-triggered immunity that protects the plant against most nonadapted pathogens (Couto and Zipfel, 2016). Conversely, pathogens try to overcome plant defenses by releasing effectors that alternatively can also be recognized by cytoplasmic receptors (Cui et al., 2015; Couto and Zipfel, 2016). Following either PAMPs or effector recognition, plant immune responses involve a complex network of signaling pathways that can be modulated by phytohormones (Pieterse et al., 2012). Salicylic acid (SA) and jasmonic acid (JA) are recognized as the two major defense hormones, and their response pathways are usually considered effective against biotrophic and necrotrophic pathogens, respectively (Pieterse et al., 2012). Other phytohormones, mainly ethylene and abscisic acid (ABA), are also involved in the defense response interacting synergically or antagonistically (Shigenaga and Argueso, 2016; Berens et al., 2017). In the case of *Arabidopsis*, other key components of the innate immune system are tryptophan-derived secondary metabolites such as the phytoalexin camalexin and the indole glucosinolates (IGs) (Bednarek, 2012). The biosynthesis of these compounds is induced in response to different pathogens, including bacteria and fungi (Clay et al., 2009; Ahuja et al., 2012), and their role in the immune response has been confirmed by analysis of different biosynthetic mutants (Tsuji et al., 1992; Glazebrook and Ausubel, 1994; Thomma et al., 1999; Lipka et al., 2005; Bednarek et al., 2009; Clay et al., 2009). It is important to note that JA has been acknowledged as a regulator of the Trp derivatives biosynthesis (Guo et al., 2013). Simultaneous applications of glucose and JA have a dramatic impact on both aliphatic and indolic glucosinolates accumulation, although the latter ones seem to be more sensitive to the treatments.

As a first step to elucidate the role of glycosylated sterols in the plant response to biotic stress, we have assayed the response of the *Arabidopsis* double mutant *ugt80A2;B1* against *Botrytis cinerea* infection, which is considered the second most important plant pathogen (Dean et al., 2012). This fungus produces several toxic compounds and cell wall degrading enzymes that can kill the host cells and decompose the plant tissue (Williamson et al., 2007). In *Arabidopsis*, global transcriptional analyses of *B. cinerea*–infected plants have identified thousands of transcripts whose expression is altered upon infection (AbuQamar et al., 2006; Birkenbihl and Somssich, 2011; Mulema and Denby, 2012; Windram et al., 2012). These data, together with genetic studies, have shown that several groups of transcription factor families, including ERFs (Huang et al., 2016; Zhang et al., 2016), WRKYs (Birkenbihl et al., 2012; Jiang and Yu, 2016), MYBs (Ramírez et al., 2011; Mengiste, 2012), and NACs (Wang et al., 2009; Nuruzzaman et al., 2013), have a major role in coordinating these changes, but only few target genes or upstream regulators have been identified (Windram et al., 2012). An exception is WRKY33, which targets multiple signaling pathways simultaneous upon *B. cinerea* infection, acting as a dual transcription factor in a promoter-dependent manner (Liu et al., 2015) because it binds directly to the promoter of genes involved in JA signaling (*JAZ1* and *JAZ5*), ET-JA crosstalk (*ORA59*), and camalexin biosynthesis (*PAD3* and *CYP71A13*) up-regulating their expression, but down-regulates the expression of other targets, as some ABA biosynthetic genes (*NCED3* and *NCED5*) (Birkenbihl et al., 2012; Liu et al., 2015). In addition, Pangesti et al. (2016) already suggested that the JA-responsive transcription factor *ORA59* is related to the camalexin accumulation during ISR.

Here we report that the *ugt80A2;B1* mutant shows increased resistance against *B. cinerea* infection, which is paralleled by an increase in the levels of JA and camalexin, and a concomitant up-regulation of several genes involved in the defense JA signaling pathway and the biosynthesis of camalexin, as well as of some of the transcription factors mentioned above, suggesting that the resistance phenotype observed in the mutant is the result of these transcriptional and metabolic changes.

## Materials and Methods

### Plant Material and Growth Conditions

All *A. thaliana* plants used in this study were of the Wassilewskija (Ws-0) ecotype. The generation of the *ugt80A2;B1* double mutant by crossing two single mutants carrying homozygous T-DNA insertions in the *UGT80A2* and *UGT80B1* genes and the subsequent characterization of the single and double mutant lines have been previously reported by DeBolt et al. (2009). Seeds of the double mutant were kindly provided by Dr. DeBolt (University of Kentucky, USA). Mutants and wild-type (WT) seeds were stratified at 4°C for 3 days and sown in *jiffy7* peat pellets (Clause-Tezier Ibérica, http://www.clausetezier.com/). Plants were grown in a chamber with a light intensity of 150 to 200 μEm^−2^ s^−1^ at 23°C under 10-h light/14-h dark cycles and 60% humidity.

### *Botrytis cinerea* Infection

For *B. cinerea* infections, six fully expanded leaves of 5-week-old plants were inoculated as described by Coego et al. (2005) with 6 ml droplets of a fungal spore suspension containing 2.5 × 10^4^ spores microliters in potato dextrose broth (PDA) (12 g L^−1^, Difco). Plants exposed to the same treatment but without fungal spores were used as control (mock). All the treated plants were covered with transparent plastic to maintain 100% relative humidity and returned to the growth chamber. Four biological replicates with 12 to 15 WT or mutant plants were performed for each treatment (infected or mock). Disease symptoms were evaluated by determining the lesion diameter of at least 50 lesions 3 days after inoculation. Three more biological replicates (15–20 plants per treatment) were performed to analyze changes in gene expression and metabolite levels (hormones and camalexin) induced by fungal infection. For this, infected or mock-treated rosette leaves from WT and mutant plants were harvested before (0 h) and after infection (24 and 48 h), pooled (five to six plants per time point and treatment), frozen in liquid nitrogen, lyophilized, and stored until used.

### High-Throughput Reverse Transcription–Quantitative Polymerase Chain Reaction Analyses of Gene Expression

Lyophilized rosette leaf samples (10 mg) from *Arabidopsis* WT and mutant plants obtained as described above were used for total RNA extraction using a Maxwell 16 LEV Plant RNA kit (Promega) and a Maxwell^®^ 16 Instrument (Promega) according to manufacturer’s instructions. The cDNA samples for reverse transcription–quantitative polymerase chain reaction (RT-qPCR) gene expression analyses were prepared from 1 microgram of total RNA using SuperScript III Reverse Transcriptase (Invitrogen) and oligo(dT) primers according to the manufacturer’s instructions. The expression of the different genes analyzed in this work was quantified by real-time PCR using the Biomark^™^ instrument (Fluidigm Corporation, San Francisco, USA) and 2 × SsoFast^™^ EvaGreen^®^ Supermix with low Rox (Bio-Rad, www.bio-rad.com) as previously reported (Manzano et al., 2016), using *PP2AA3* (At1g13320) (Hong et al., 2010) and *UBC* (At5g25760) (Czechowski et al., 2005) as housekeeping reference genes and specific primers for each analyzed gene (**Supplemental Table 1**). Data for each WT and *ugt80A2;B1* mutant samples, infected or treated with mock, are expressed as normalized quantity values versus the housekeeping genes. Expression was calculated using Data Analysis Gene Expression software (http://www.dagexpression.com/dage.zip) (Ballester et al., 2013). Quantification of transcript levels was done in three independent biological replicates, and for each biological replicate, two technical replicates were performed.

### Determination of Hormones and Camalexin Levels

Hormones and camalexin were extracted from the same samples used for gene expression analysis as described by Sánchez-Bel et al. (2017). Briefly, 30 mg of dry material was extracted with 1 ml of H_2_O:MeOH (90:10) with 0.01% of HCOOH with a mix of internal standards. After centrifugation and filtration of the supernatant with 0.22-µm filter of regenerated cellulose, 20 µl was injected into a Waters Acquity UPLC coupled with a triple quadrupole tandem mass spectrometer (Waters), and the separation of compounds was performed with a Kinetex C18 analytical column (Phenomenex), 5 µm of particle size and 2.1 × 100 mm. Before the analysis, external calibration curves with pure chemical standards were obtained for each tested compound complemented with heavy isotopes of each hormone as internal standards. The MassLynx 4.1 software (Waters) was used to process the quantitative data from calibration standards and plant samples.

## Results

### Impairment of SGs Biosynthesis Leads to Enhanced Resistance of *Arabidopsis* to *B. cinerea* Infection

**Figure 1:**
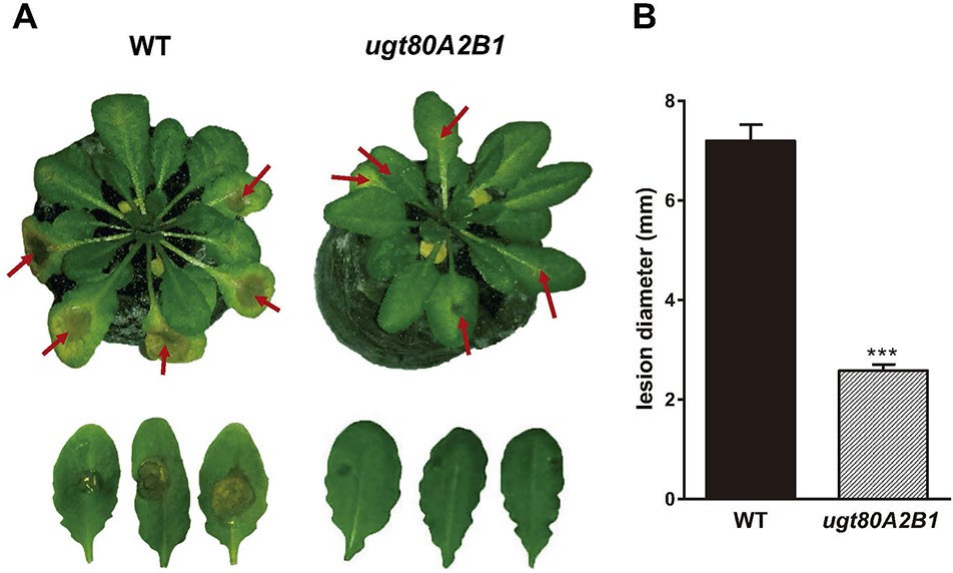
The *ugt80A2;B1* mutant impaired in steryl glycosides biosynthesis shows enhanced resistance to *B. cinerea* infection. **(A)** Symptoms of infection in leaves of wild-type (WT) and *ugt80A2;B1* mutant plants 3 days after inoculation with *B. cinerea.* Red arrows point to *B. cinerea* inoculation sites **(B)** diameter of the resulting lesions. Data represent the average ± SEM of at least 50 lesions in one experiment. The experiment was repeated three more times with similar results. Asterisks indicate significant differences between WT and mutant plants according to Student *t* test (***P < 0.001).

**Figure 2:**
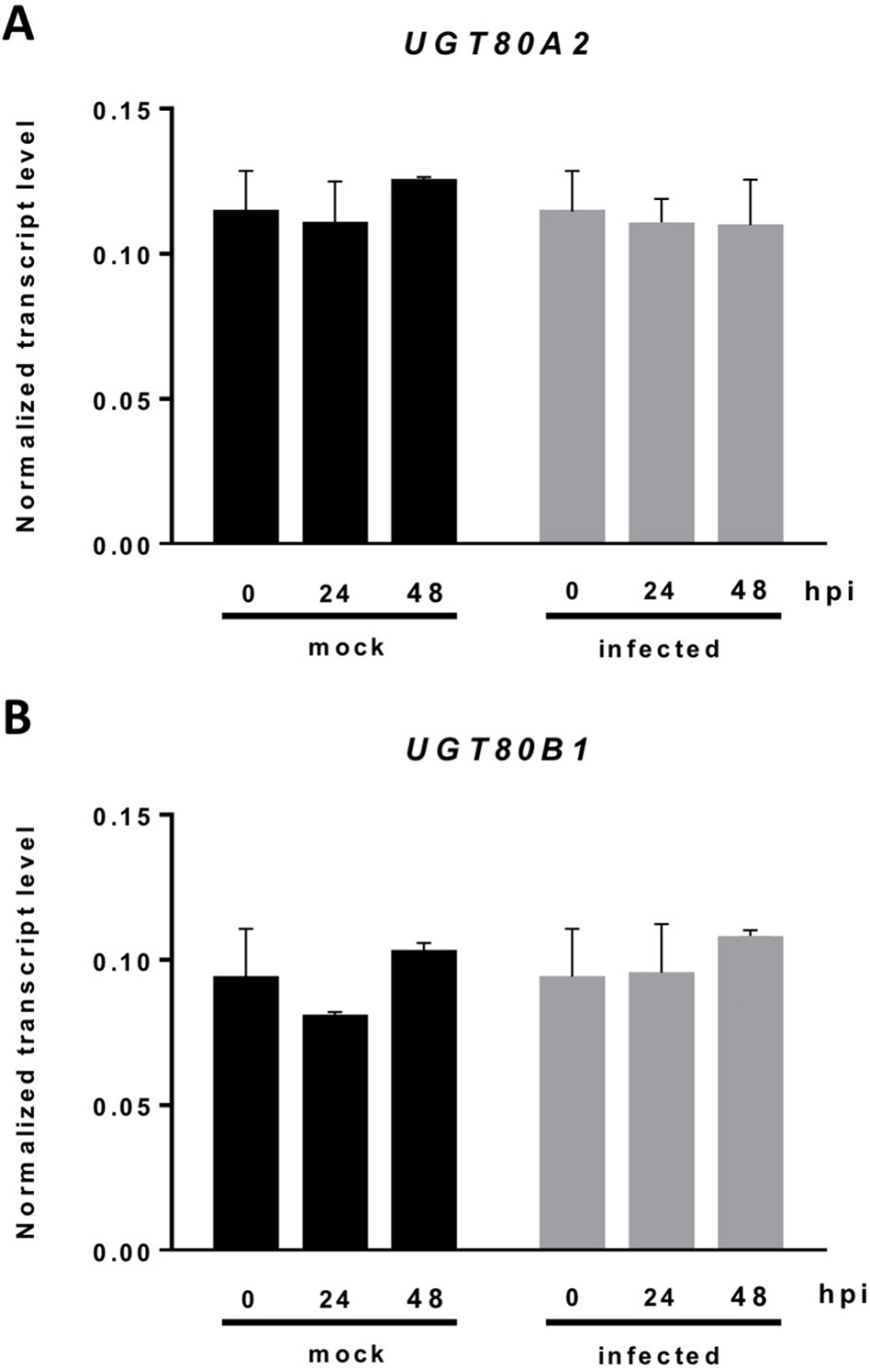
Expression of the *UGT80A2* and *UGT80B1* genes remains unchanged upon *B. cinerea* infection. The transcript levels of *UGT80A2* **(A)** and *UGT80B1* **(B)** were measured by reverse transcription–quantitative polymerase chain reaction using RNA extracted from rosette leaves of *Arabidopsis* WT plants inoculated (infected) or not (mock) with *B. cinerea* at time points 0, 24, and 48 h. Data for each WT sample, infected or treated with mock, are expressed as normalized quantity values using two independent housekeeping genes (*UBC* and *PP2A*). Values are means ± SEM of three independent biological experiments.

The current knowledge about the specific contribution of glycosylated sterols to plant biotic stress response is scarce. To gain some insight about the role of these compounds in the plant response to this kind of stress, we checked the effect of *B. cinerea* infection, a common necrotrophic fungal pathogen, in *Arabidopsis* WT plants (Ws-0) and the previously generated double mutant *ugt80A2;B1*, which has inactivated the two genes reported to encode SGT in *Arabidopsis* (*UGT80A2* and *UGT80B1*) and presents reduced levels of glycosylated sterols in different plant organs, including the rosette leaves (DeBolt et al., 2009). To this end, leaves of WT and mutant plants were inoculated with a *B. cinerea* spore suspension, and the size of the resulting lesions was measured 3 days after inoculation. The results from four independent experiments showed that the average diameter of the lesions in the *ugt80A2;B1* mutant plants was significantly smaller (about one half) than in the WT plants (**Figure 1**). These results indicate that the simultaneous inactivation of *Arabidopsis UGT80A2* and *UGT80B1* genes results in increased resistance against *B. cinerea* infection. Interestingly, infection with this necrotrophic fungus did not affect the expression of *UGT80A2* and *UGT80B1* genes in the WT plants because their transcript levels at 24 and 48 h postinoculation (hpi) remained unchanged compared to the noninfected plants (**Figure 2**).

### Resistance of the *Arabidopsis ugt80A2;B1* Mutant to *B. cinerea* Involves JA Signaling

**Figure 3:**
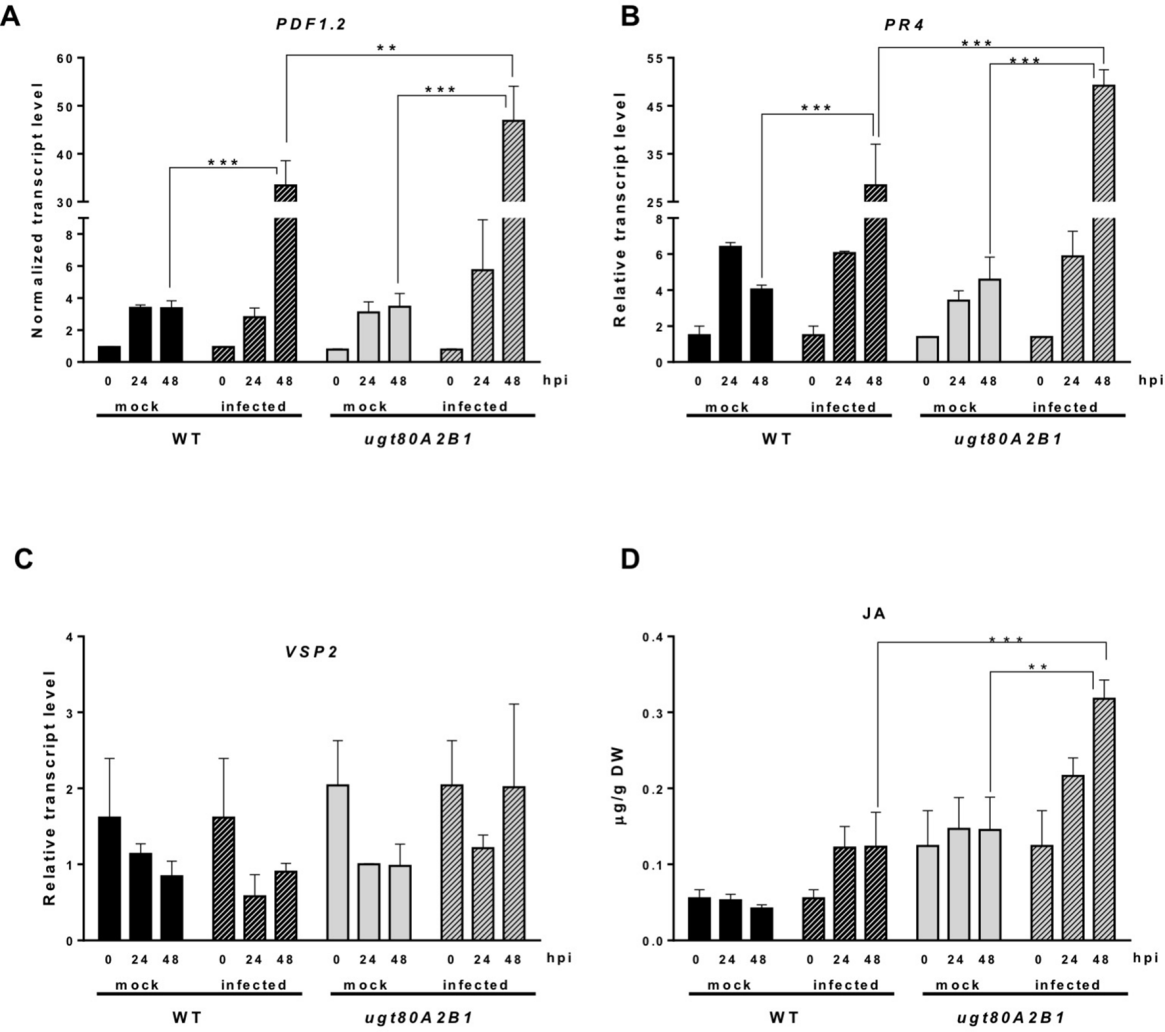
Increased transcript levels of JA-responsive marker genes and JA levels in *ugt80A2;B1* mutant plants compared to WT upon infection with *B. cinerea*. The transcript levels of *PDF1.2* **(A)**, *PR4* **(B)**, and *VSP2* **(C)** were determined by reverse transcription–quantitative polymerase chain reaction using RNA extracted from rosette leaves of *Arabidopsis* WT and *ugt80A2;B1* mutant plants infected or not (mock) with *B. cinerea* at time points 0, 24, and 48 h. Data for each WT and *ugt80A2;B1* mutant samples, infected or treated with mock, are expressed as normalized quantity values calculated using two independent housekeeping genes (*UBC* and *PP2A*). JA was quantified in leaf extracts by ultraperformance liquid chromatography–mass spectrometer and expressed as µg/g of dry weight **(D)**. Values are means ± SEM of three independent biological experiments. Asterisks represent significant differences determined by one-way analysis of variance (**P < 0.005, ***P < 0.001).

The JA-mediated defense pathway is assumed to have a central role in plant resistance against necrotrophic pathogens (Rowe et al., 2010). In order to determine if the resistance to the *B. cinerea* observed in the *ugt80A2;B1* mutant was associated to this pathway, we analyzed the expression of some JA-responsive marker genes of the two major branches recognized in the *Arabidopsis* JA signaling pathway, the ERF and the MYC branches (Pieterse et al., 2012), in plants infected or not with the pathogenic fungus at different time points. The expression of *PDF1.2*, a JA-responsive gene representative of the ERF branch, was significantly induced after infection with *B. cinerea* both in the WT and the mutant plants, but at 48 hpi, the induction in the *ugt80A2;B1* mutant was about twice that in the WT plants (**Figure 3A**). A similar expression pattern was observed for *PR4*, another JA-responsive gene of the ERF branch, but in this case, the transcript levels were more than twofold higher in the mutant than in the WT (**Figure 3B**). On the contrary, the expression of the MYC-branch representative gene *VSP2* was not significantly affected by the infection neither in the WT plants nor in the mutant (**Figure 3C**). This was not unexpected because activation of the MYC branch has been related with defense against chewing insects, while defense against necrotrophic pathogens is mediated by the ERF one (Pieterse et al., 2012). In addition to genetic responses, plants usually experience important hormonal changes after pathogen attack. Thus, we measured the levels of JA in the same tissue samples used for the gene expression analysis. As shown in **Figure 3D**, JA levels increased after infection with the fungal pathogen in both WT and *ugt80A2;B1* mutant plants. However, JA levels were markedly higher in the mutant than in the WT, with values that were approximately twofold and threefold higher at 24 and 48 hpi, respectively (**Figure 3D**). It is worth to mention that 48 h after *B. cinerea* infection the expression of *ACS6*, a gene involved in ethylene biosynthesis (Li et al., 2012), increased more than twofold in the WT plants and about fourfold in the *ugt80A2;B1* mutant compared with the mock treatment at the same time point (**Figure S1**). This hormone interacts synergistically with JA in the ERF branch (Pieterse et al., 2012). However, the expression of *NCED3* and *RAB18*, two genes involved, respectively, in the biosynthesis and response to ABA, a hormone that interacts with JA in the MYC branch (Anderson et al., 2004), was not affected by *B. cinerea* treatment neither in the WT nor in the mutant plants (**Figure S2**).These results indicate that the resistance of the *ugt80A2;B1* mutant to *B. cinerea* is mediated by the ERF branch of the JA pathway, mainly as a result of an increased accumulation of this hormone in the infected mutant.

A crosstalk between hormone signaling pathways, particularly those mediated by SA and JA, has been found to contribute to plant resistance to different types of pathogens (Pieterse et al., 2012). Therefore, SA levels were determined in the same samples used for JA quantification. The levels of SA were similar in WT and *ugt80A2;B1* mutant plants, and no significant changes were detected upon infection (**Figure S3A**). Furthermore, significant differences were neither observed between the WT and the mutant plants when the expression levels of *NPR1*, the gene encoding the main regulatory protein of the SA signaling pathway, were determined in plants infected or not with the pathogen (**Figure S3B**). The expression of *PR1*, a marker gene of the SA signaling pathway, increased about 10-fold upon fungus infection (48 hpi) either in the WT or in the *ugt80A;2B1* mutant plants (**Figure S3C**). These results suggest that the SA-mediated defense pathway is not involved in the response of the *ugt80A2;B1* mutant to *B. cinerea *infection.

### The Synthesis of Camalexin and Indole Glucosinolates Is Induced in the *ugt80A2;B1* Mutant Upon *B. cinerea* Infection

**Figure 4:**
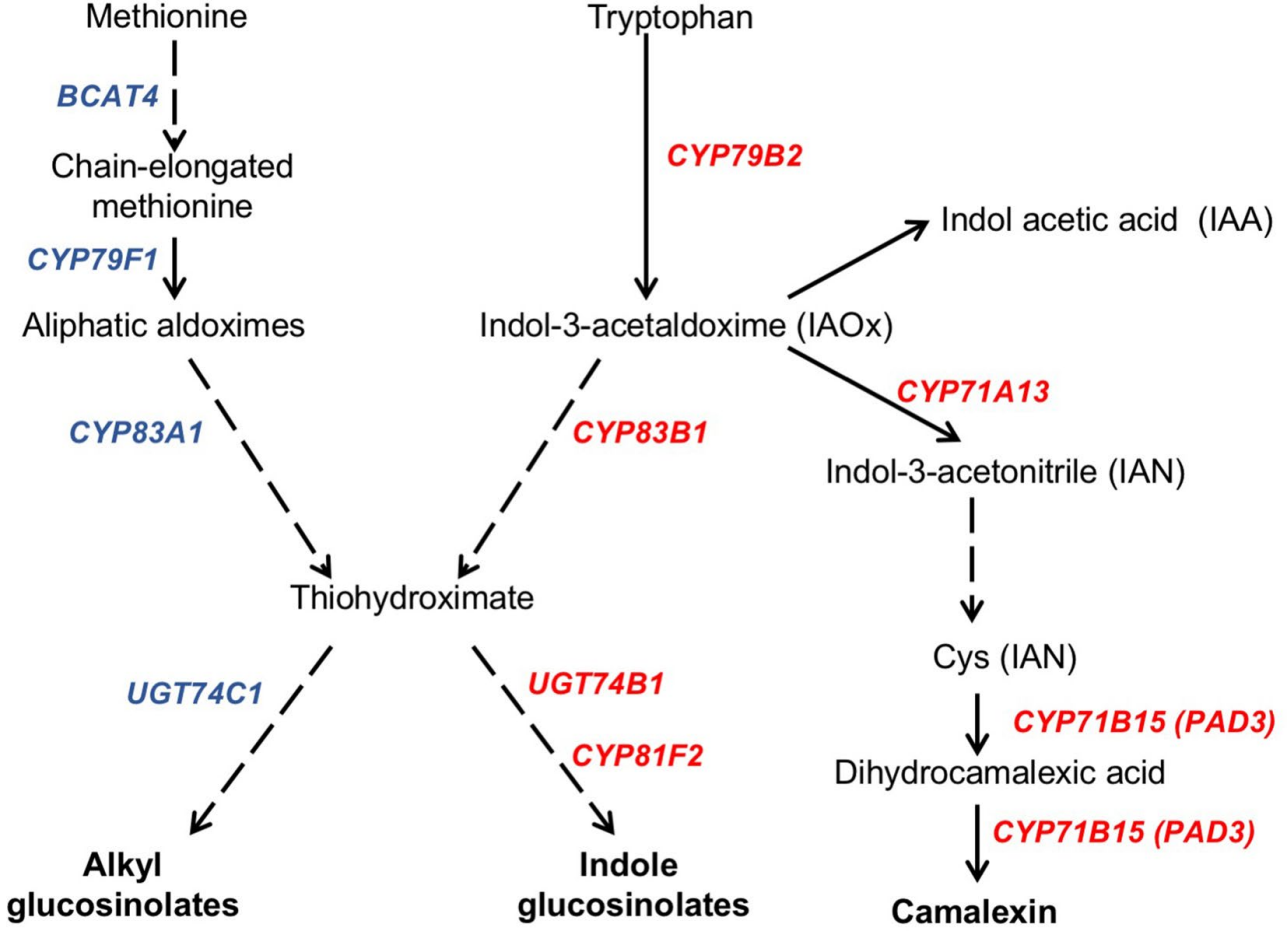
Schematic representation of camalexin and glucosinolate biosynthesis pathways. The biosynthesis of the tryptophan (camalexin, indole glucosinolates, and IAA) and methionine (alkylglucosinolates) derived compounds is indicated in a simplified form showing the biosynthetic steps mediated by genes whose expression levels have been quantified in this work. Genes whose expression increase in the *ugt80A2;B1* mutant compared to WT upon *B. cinerea* infection are indicated in red, whereas those whose expression does not change are shown in blue. Solid arrows indicate single enzymatic steps, whereas dashed ones represent several enzymatic steps. Figure is based on previous representations of these pathways (Kliebenstein et al., 2005; Yatusevich et al., 2010; Frerigmann et al., 2016).

**Figure 5:**
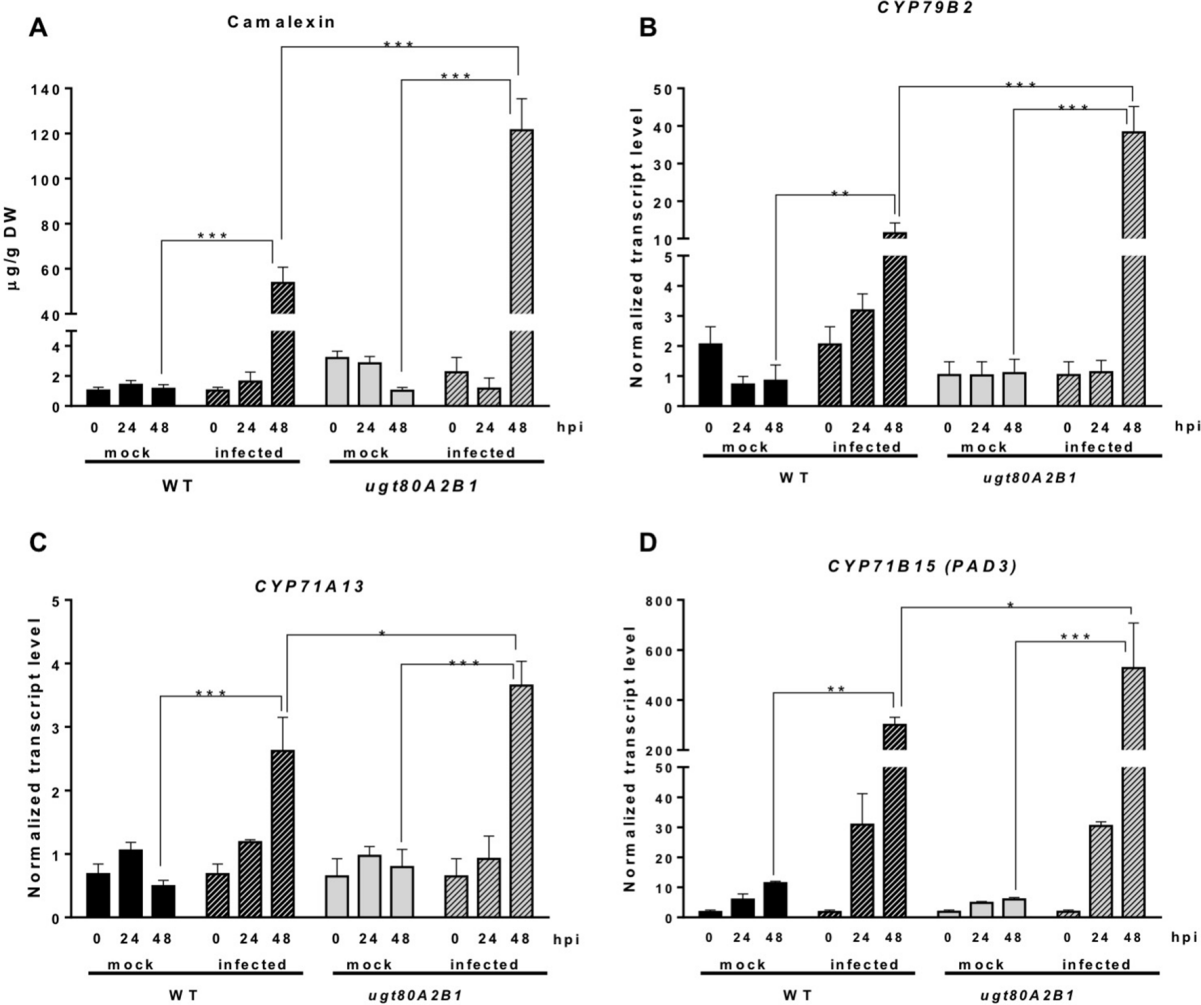
The *ugt80A2;B1* mutant displays camalexin accumulation and increased transcript levels of camalexin biosynthetic genes in comparison to WT upon infection with *B. cinerea*. Camalexin was quantified in leaf extracts of *Arabidopsis* WT and *ugt80A2;B1* mutant plants infected or not (mock) with *B. cinerea* at time points 0, 24, and 48 h using ultraperformance liquid chromatography–mass spectrometer and is expressed as µg/g of dry weight **(A)**. The transcript levels of *CYP79B2* **(A)**, *CYP71A13* **(B)** and *CYP71B15 (PAD3)* **(C)** were determined by reverse transcription–quantitative polymerase chain reaction using RNA extracted from the same leaf samples used for camalexin quantification. Data for each WT and *ugt80A2;B1* mutant samples, infected or treated with mock, are expressed as normalized quantity values calculated using two independent housekeeping genes (*UBC* and *PP2A*). Values are means ± SEM of three independent biological replicates. Asterisks represent significant differences determined by one-way analysis of variance (*P < 0.05, **P < 0.005, ***P < 0.001).

In response to pathogen attack, plants induce the biosynthesis of phytoalexins and other defense secondary metabolites, such as glucosinolates (**Figure 4**). Because camalexin is the main phytoalexin accumulated in *Arabidopsis* after infection by fungi or bacteria, and its biosynthesis has been reported to be elicited by JA (De Geyter et al., 2012), we investigated if it could be involved in the resistance response observed in the *Arabidopsis ugt80A2;B1* mutant infected with *B. cinerea*. To this end, the levels of camalexin were analyzed in the WT and mutant plant samples used for JA quantification. A marked accumulation of this compound was detected in WT and *ugt80A2;B1* plants after 48 hpi with *B. cinerea*, but the levels in the mutant were significantly higher (about twofold) than in the WT (**Figure 5A**). The accumulation of camalexin in response to fungal infection was paralleled by an increase in the expression of several genes related to its biosynthesis (**Figures 5B–D**). The expression of the *CYP79B2*, *CYP71A13*, and *CYP71B15* biosynthetic genes was strongly induced by fungal infection, particularly at 48 hpi, both in the WT and the *ugt80A2;B1* mutant plants, but the transcript levels of these three genes were higher in the mutant than in the WT plants, specifically about threefold in the case of *CYP79B2* (**Figure 5B**) and approximately 1.5-fold in *CYP71A13* and *CYP71B15* (**Figures 5C, D**).

**Figure 6:**
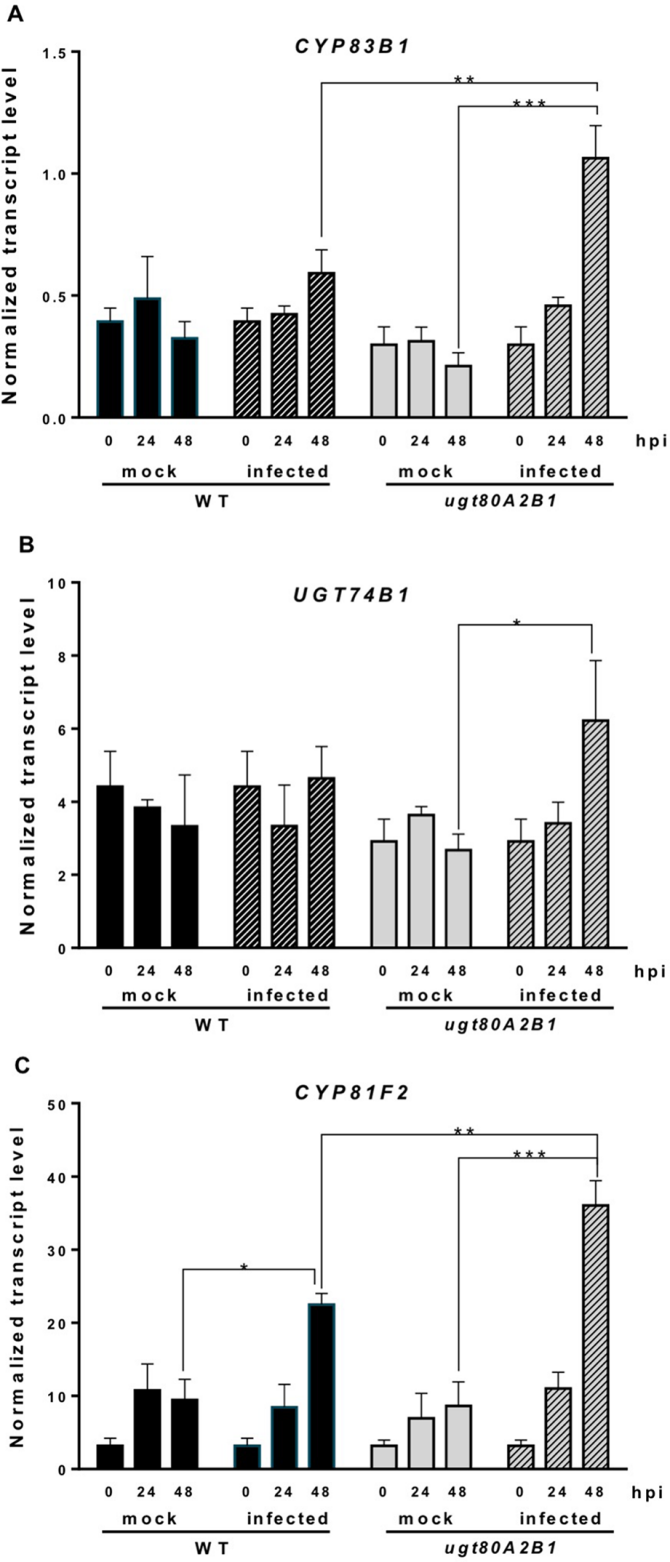
Increased transcript levels of indole glucosinolate biosynthetic genes in the *ugt80A2;B1* mutant compared to WT upon infection with *B. cinerea*. The transcript levels of *CYP83B1* **(A)**, *UGT74B1* **(B)**, and *CYP81F2* **(C)** were determined by reverse transcription–quantitative polymerase chain reaction using RNA extracted from rosette leaves of *Arabidopsis* WT and *ugt80A2;B1* mutant infected or not (mock) with *B. cinerea* at different time points (0, 24, and 48 h). Data for each WT and *ugt80A2;B1* mutant samples, infected or treated with mock, are expressed as normalized quantity values calculated using two independent housekeeping genes (*UBC* and *PP2A*). Values are means ± SEM of three independent biological replicates. Asterisks represent significant differences determined by one-way analysis of variance (*P < 0.05, **P < 0.005, ***P < 0.001).

**Figure 7:**
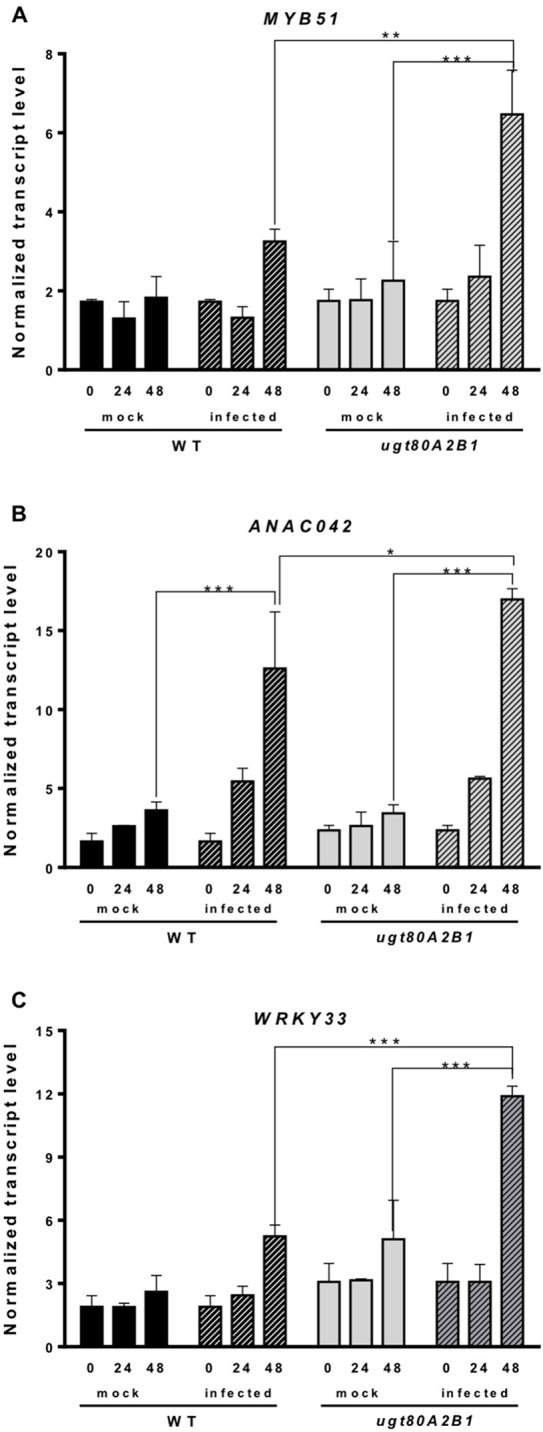
The *ugt80A2;B1* mutant shows increased transcript levels of genes coding for transcription factors regulating camalexin and indole glucosinolate biosynthesis compared to WT upon infection with *B. cinerea*. The transcript levels of *MYB51* **(A)**, *ANAC042* **(B)** and *WRKY33* **(C)** were determined by reverse transcription–quantitative polymerase chain reaction using RNA extracted from rosette leaves of *Arabidopsis* WT and *ugt80A2;B1* mutant infected or not (mock) with *B. cinerea* at different time points (0, 24, and 48 h). Data for each WT and *ugt80A2;B1* mutant samples, infected or treated with mock, are expressed as normalized quantity values calculated using two independent housekeeping genes (*UBC* and *PP2A*). Values are means ± SEM of three independent biological replicates. Asterisks represent significant differences determined by one-way analysis of variance (*P < 0.05, **P < 0.005, ***P < 0.001).

Camalexin biosynthesis involves the conversion of tryptophan to indole-3-acetaldoxime (IAOx), which is also the precursor of the phytohormone indole-3-acetic acid (IAA) and the plant defense secondary metabolites IGs (**Figure 4**). Thus, we investigated if the inactivation of the two *Arabidopsis* SGTs also affected these biosynthetic pathways. While no relevant changes were observed in the IAA levels between WT and mutant plants (**Figure S4**), the transcript levels of several genes encoding specific enzymes of the indole glucosinolate pathway, such as *CYP83B1*, *UGT74B1*, and *CYP81F2* (Sønderby et al., 2010), were higher in the mutant than in the WT plants at 48 hpi (**Figure 6**). The expression of *CYP83B1* and *UGT74B1* remained essentially unaltered in noninoculated leaves but was significantly up-regulated 48 h after *Botrytis* inoculation only in the *ugt80A2;B1* mutant (**Figures 6A, B**). The transcript levels of *CYP81F2*, a gene specifically involved in the synthesis of 4-hydroxy-3-indolyl-methyl glucosinolates, increased significantly in the WT and mutant plants at 48 hpi, but this increase was significantly higher in the mutant than in control plants (**Figure 6C**). Altogether these data indicate that a transcriptional activation of the pathways involved in the synthesis of the Trp-derived defense compounds camalexin and indole glucosinolates is induced in the *Arabidopsis ugt80A;2B1* mutant upon infection with *B. cinerea*. In agreement with the above results, the expression of some genes encoding transcriptional regulators of the camalexin and indole glucosinolates biosynthetic genes in the infected mutant was higher than in the infected WT plants. As shown in **Figure 7A**, the expression of the *MYB51* transcription factor, a positive regulator of the biosynthetic steps required for the production of IAOx (Frerigmann et al., 2015), was significantly more expressed at 48 hpi in the *ugt80A2;B1* mutant than in the WT plants, while the expression of *ANAC042*, a regulator of camalexin biosynthesis that acts downstream IAOx (Saga et al., 2012), increased in both WT and *ugt80A2;B1* mutant plants upon infection, but at 48 hpi, this increase was significantly higher in the mutant (**Figure 7B**). A similar induction profile was observed for the transcript levels of *WRKY33* (**Figure 7C**), a transcription factor activated by the mitogen-activated protein kinase cascade that has been well characterized as a camalexin biosynthesis inductor (Saga et al., 2012). All these results suggest that camalexin and, probably, also indole glucosinolates are actively involved in the enhanced resistance of the *ugt80A2;B1* mutant to *B. cinerea* infection.

### The Synthesis of Alkylglucosinolates in the *Arabidopsis ugt80A2;B1* Mutant Is Not Affected by *B. cinerea* Infection

Alkylglucosinolates (AGs) are also a class of plant defense secondary metabolites whose biosynthetic pathway is related to that of indole glucosinolates (**Figure 4**), and it has been reported that both biosynthetic pathways may affect each other (Liu et al., 2016). This prompted us to investigate if the biosynthesis of this kind of glucosinolates could also be altered in the *ugt80A2;B1 Arabidopsis* mutant. To check this possibility, we analyzed the expression of several genes encoding enzymes involved in their biosynthetic pathway. The transcript levels of *BCAT4* and *CYP79F1* were similar in the WT and *ugt80A2;B1* mutant plants infected or not with *B. cinerea* (**Figures S5A, B**). These genes are involved in the first stages of AGs biosynthesis, which involves the side-chain elongation of the precursor amino acid methionine and its subsequent oxidation to aldoxime (Sønderby et al., 2010) (**Figure 4**). No significant changes were observed between WT and mutant plants in the transcript levels of *CYP83A1* (**Figure S5C**) and *UGT74C1* (**Figure S5D**), two genes involved, respectively, in the metabolism of the aldoxime to the corresponding alkylthiohydroximate and the subsequent conversion of this intermediate to AGs (**Figure 4**). These results indicate that, unlike indole glucosinolates, the synthesis of AGs is not transcriptionally activated either in the *ugt80A2;B1* mutant or in the WT upon infection with *B. cinerea*. This observation is further supported by the lack of induction of *MYB28* and *MYB29*, two genes coding for transcription factors that positively regulate the expression of many alkyl glucosinolate biosynthetic genes (Yatusevich et al., 2010) whose transcript levels are similar in the WT and the mutant plants infected or not with the pathogen (**Figures S5E, F**). These results suggest that AGs are not involved in the defense response of *Arabidopsis* against infection by *B. cinerea* whether plants have normal or depleted levels of glycosylated sterols.

## Discussion

Changes in the profile of glycosylated sterols have been widely related with the plant response to different abiotic stresses (Palta et al., 1993; Mishra et al., 2013; Pandey et al., 2014; Mishra et al., 2015; Saema et al., 2016; Takahashi et al., 2016; Singh et al., 2017). However, there are less experimental evidence supporting their involvement in biotic stress responses (Pandey et al., 2014; Singh et al., 2016; Mishra et al., 2017). Furthermore, most of the published data related with this issue were obtained using Solanaceae species with simultaneously altered levels of glycosylated sterols and glycosylated defense compounds, such as whithanolides in *W. somnifera* (Singh et al., 2016) and rutin in tobacco (Pandey et al., 2014), which could be ultimately responsible for the observed responses. Because *A. thaliana* lacks this kind of specialized secondary metabolites, it represents a suitable model to assess the role of conjugated sterols in plant defense against biotic agents. In this regard, Kopischke et al. (2013) reported a role for conjugated sterols in the plant response to *Phytophthora infestans* using the *Arabidopsis psat1* mutant impaired in steryl ester (SE) biosynthesis. However, the observed response could not be correlated with changes in a specific sterol fraction because the levels of SE and ASG are reduced in the leaves of this mutant, whereas those of SGs are increased, and FSs remain unaltered. Because *Phytophthora* is a sterol-auxotroph pathogen, the altered profile of sterols in the mutant, together with the described capacity of pathogenesis-related 1 (PR-1) to inhibit pathogen growth by sequestering its sterols (Gamir et al., 2017), might explain the resistance phenotype of the *psat1* mutant. Our results suggest that reduced levels of glycosylated sterols in the *Arabidopsis ugt80A2;B1* mutant (DeBolt et al., 2009) confer resistance to the necrotrophic fungus *B. cinerea* (**Figure 1**). The leaves of this mutant contain normal levels of nonglycosylated sterols (FSs + SE), whereas those of glycosylated sterols (SG + ASG) are markedly reduced, albeit not completely abolished (DeBolt et al., 2009). Thus, our results establish for the first time a direct link between reduced levels of glycosylated sterols and resistance against pathogen attack. We also show that expression of the two genes encoding the SGTs that synthesize the bulk of SGs in *Arabidopsis* remains unaltered upon infection with *B. cinerea* (**Figure 2**). This observation supports the notion that SG biosynthesis is not induced in response to *B. cinerea* infection, although the possibility that a gene coding for an as yet unreported SGT potentially involved in the residual production of SGs and/or the synthesis of a specialized SG could be up-regulated cannot be entirely excluded. It is reported that although both enzymes display sterol glucosyltransferase activity, substrate specificity is apparent in that UGT80A2 is responsible for the accumulation of major SGs, while UGT80B1 is involved in accumulation of minor SGs and ASGs (Stucky et al., 2015).

In order to understand the molecular mechanism acting behind the resistance phenotype observed in the *ugt80A2;B1* mutant plants, we first measured the levels of JA and SA in mutant and WT plants infected or not with *B. cinerea* because it is well known that these phytohormones act as primary signals in the regulation of plant responses to biotic stress (Santino et al., 2013). After infection with *B. cinerea*, JA content increased in both WT and mutant plants (**Figure 3D**), which is not surprising because an increase in the levels of this hormone has long been described in response to necrotrophic pathogen infection (Penninckx et al., 1996) and herbivore damage (Reymond et al., 2000). However, after 24 and 48 hpi, the JA levels were significantly higher in the mutant than in the WT (**Figure 3D**), and this differential increase correlated with a stronger up-regulation of some defense genes such as *plant defensin 1.2* (*PDF1.2*) and the *pathogenesis related protein 4* (*PR4*) in the infected *ugt80A;2B* mutant compared to WT (**Figures 3A, B**). These two genes are markers of the ERF branch of the JA signaling pathway that is activated upon necrotrophic pathogen attack (Santino et al., 2013), suggesting that this branch of the downstream JA signaling is activated in the *ugt80A;2B* mutant after *Botrytis* infection. Interestingly, the expression of *ACS6* increased about twofold and fourfold, respectively, in WT and *ugt80A2;B1* mutant plants 48 h after fungus infection (**Figure S1**), which agrees with its previously reported induction by *B. cinerea* infection (Han et al., 2010; Li et al., 2012). *ACS6* is one of the nine members of the *Arabidopsis* gene family encoding 1-amino-cyclopropane-1-carboxylic acid synthase, the rate-limiting enzyme in ethylene biosynthesis (Wang et al., 2002), a hormone that acts synergistically with JA on the expression of the ERF branch signaling pathway upon infection by necrotrophic pathogens (Pieterse et al., 2012). On the contrary, the JA signaling branch regulated by the *MYC2* transcription factor, which has been reported to have a specific role in response to insect attack (Santino et al., 2013), was not activated after *Botrytis* infection neither in the WT nor in the mutant plants because no significant changes were observed in the expression of the MYC-branch marker gene *vegetative storage protein 2* (*VSP2*) (**Figure 3C**). This is in accordance with the absence of changes observed after fungus infection in the expression of *NCED3*, one of the major genes encoding 9-*cis*-epoxycarotenoid dioxygenase, a key enzyme in the biosynthesis of ABA (Leng et al., 2014), and *RAB18*, an ABA-responsive gene, either in the WT or the *ugt80A2;B1* mutant plants (**Figure S2**), because ABA has been reported to act synergistically with JA on the expression of the MYC branch upon wounding or herbivory attack (Anderson et al., 2004). Similarly, the levels of SA, a hormone that usually interacts antagonistically with JA (Pieterse et al., 2012), and the expression of the *NPR1* gene coding for a key transcriptional activator of the SA-dependent immune response (Pieterse et al., 2012) remained unaltered in infected and noninfected WT and mutant plants (**Figures S3A, B**). The expression of *PR1*, a marker gene of SA response (Pieterse et al., 2012), increased about 10-fold either in WT or mutant plants upon *Botrytis* infection (**Figure S3C**). This agrees with the results of Govrin and Levine (2002) indicating that *PR1* induction by *B. cinerea* can be independent of SA. These results indicate that the SA signaling pathway is not involved in the response to *B. cinerea* either in the WT or in the mutant plants. All these observations indicate that depletion of glycosylated sterols content in the *Arabidopsis ugt80A2;B1* mutant leads to an enhancement of *Botrytis*-induced JA levels that specifically activate the JA signaling pathway regulated by the ERF family of transcription factors. This finding further reinforces the hypothesis that changes in the relative proportions of sterols are perceived by plants as a stress signal that activates different hormone-related defensive responses in a sterol profile-dependent manner (Wang et al., 2012; Singh et al., 2015; Manzano et al., 2016).

The intricate immune response network evolved by plants to protect themselves against pathogens includes also the biosynthesis of different types of secondary metabolites that serve as defense compounds, such as phytoalexins and glucosinolates. Camalexin, a tryptophan-derived compound (**Figure 4**), is the major phytoalexin of *A. thaliana* (Glawischnig, 2007). Interestingly, camalexin content was enhanced by *B. cinerea* infection in both WT and *ugt80A2;B1* mutant plants (**Figure 5A**), but their levels were significantly higher in the mutant than in the WT plants (**Figure 5A**), suggesting a role of this phytoalexin in the resistance to the fungus observed in the mutant. A positive role of camalexin in plant resistance against pathogens, including several necrotrophic fungi, has previously been demonstrated by genetic approaches (Lemarié et al., 2015). Mutants with reduced camalexin levels show increased susceptibility to *B. cinerea*, while its accumulation has been correlated with resistance to the fungus (Ferrari et al., 2003; Kliebenstein et al., 2005; Ferrari et al., 2007; Van Baarlen et al., 2007). Because the secondary metabolites are derived from primary metabolic pathways, their biosynthesis should be temporally and spatially coordinated to maintain normal growth and plant development. Thus, the accumulation of camalexin in the proximity of the lesions induced by *Botrytis* is associated to a strong induction of tryptophan and camalexin biosynthetic genes in the same tissues (Schuhegger et al., 2006; Schuhegger et al., 2007). According to these observations, the increase in camalexin levels observed at 48 hpi (**Figure 5A**) correlates with significantly higher transcript levels of a set of cytochrome P450 genes encoding key enzymes of the camalexin biosynthetic pathway, such as *CYP79B2*, *CYP71A13*, and *CYP71B15 (PAD3)* (**Figures 5B–D**), and the *WRKY33* and *ANAC042* genes coding for transcriptional activators of camalexin biosynthesis (**Figures 7B, C**), being all these responses significantly more intense in the *ugt80A;2B1* mutant than in WT plants (**Figures 5** and **7**). It has been reported that WRKY33 binds to the promoters of *CYP71B15* and *CYP71A13* to induce camalexin biosynthesis (Petersen et al., 2008) during the early stages of pathogen infection (Birkenbihl et al., 2012), while upon induction of camalexin biosynthesis by treatment with AgNO_3_, the time course of *ANAC042* expression parallels that of the biosynthetic genes *CYP79B2*, *CYP71A12*, and *CYP71B15* (Saga et al., 2012). These observations indicate that camalexin biosynthesis induction in *Arabidopsis* leaves infected with *B. cinerea* is coordinately controlled at the transcriptional level similarly to what has been described in *Arabidopsis* roots treated with Flg22, where induction of camalexin biosynthesis was associated with the transcriptional induction of the *PAD3*, *CYP71A12*, and *CYP71A13* biosynthetic genes (Millet et al., 2010). Moreover, these results support the hypothesis that the enhanced camalexin accumulation in the *ugt80A2;B1* mutant infected with *B. cinerea* is due to a higher transcriptional up-regulation of its biosynthetic pathway compared to WT plants. Interestingly, *ANAC042*, *CYP79B2*, *CYP71A12*, and *CYP71B15* genes have been previously included in a coexpression module closely related with another module comprising, among others, *MYB51* (Saga et al., 2012), a gene reported to encode a positive regulator of both camalexin and IG biosynthesis whose expression is induced by *B. cinerea* infection (Frerigmann et al., 2015). Our results support these observations because the expression profile of *MYB51* (**Figure 7A**) was similar to that of *ANAC042* (**Figure 7B**), which, as mentioned above, correlated with that of some camalexin biosynthetic genes (**Figure 5**).

Indole glucosinolates are small secondary metabolites involved in plant immunity (Bednarek et al., 2009; Clay et al., 2009) that share with camalexin the initial step of their biosynthetic pathways, the conversion of tryptophan to IAOx catalyzed by CYP79B2 (**Figure 4**). MYB51, together with MYB122 and MYB34, regulates the IG biosynthesis in *A. thaliana* (Celenza et al., 2005; Gigolashvili et al., 2007), although the contribution of each MYB factor to IG production is different in shoots and roots, being MYB51 the main regulator in shoots (Frerigmann and Gigolashvili, 2014). In *Arabidopsis* WT and *ugt80A2;B1* mutant plants, the expression of *MYB51* increased after *Botrytis* infection, and its transcript levels were significantly higher in the mutant (**Figure 7A**). A similar expression pattern was observed for the genes involved in IG (*CYP83B1*, *UGT74B1*, and *CYP81F2*) (**Figure 6**) and camalexin biosynthesis (*CYP79B2, CYP71A13*, and *CYP71B15*) (**Figures 5B–D**), which supports a role of MYB51 as a transcriptional regulator of the pathways leading to the synthesis of these kinds of defense compounds. In the case of camalexin biosynthesis, MYB51 would act in concert with WRKY33 and ANAC042 to activate the entire pathway because it is known that MYB51 induces the expression of *CYP79B2* but not that of the downstream biosynthetic genes (Frerigmann et al., 2015), which as stated above would be activated by WRKY33 and ANAC042. Our results indicate that *B. cinerea* infection activates the expression of different *Arabidopsis* transcription factors (MYB51, WRKY33, and ANAC042) to enable camalexin biosynthesis. The higher expression of the genes coding for these transcriptional activators and the resulting higher accumulation of camalexin in the *ugt80A2;B1* mutant compared to WT plants could be the reason of its resistance phenotype. Because the expression of the genes involved in the IG biosynthesis is also up-regulated in the infected mutant, it is reasonable to speculate that these compounds play also a role in this defense response. It is worth to mention that MYB51 is inducible by the ERF1 branch of the JA signaling pathway (Millet et al., 2010) whereas glucosinolate levels are reduced when the JA signaling is blocked (Mikkelsen et al., 2003; Mewis et al., 2005; Li et al., 2006). This, together with the fact that *WRKY33* and *ANAC042*, can be regulated by JA (De Geyter et al., 2012) suggests that the different signaling pathways leading to the resistance phenotype against *B. cinerea* observed in the *ugt80A2;B1* mutant might be activated by the increased JA levels detected in the mutant after infection with the fungus, compared to the WT (**Figure 3D**).

Alkylglucosinolates are a class of glucosinolates synthesized from methionine that are biosynthetically related with IGs because they share the common metabolic intermediate thiohydroxymate (**Figure 4**). In fact, a crosstalk between both pathways has been reported. For instance, a *cyp83a1* mutant produces lower levels of AG, but accumulates higher levels of IG than the corresponding WT (Hemm et al., 2003; Naur et al., 2003; Sønderby et al., 2010). However, in our experimental conditions, this kind of interaction does not seem to occur since, in contrast to the changes observed in the expression of the genes coding for the IG biosynthetic enzymes and the corresponding transcriptional activators (**Figures 6** and **7**), no changes were detected between *ugt80A2;B1* mutant and WT plants infected or not with *B. cinerea* either in the expression of the *BCAT4*, *CYP79F1*, CYP83A1, and *UGT74C1* genes involved in AG biosynthesis (**Figures S5A–D**) or in the transcript levels of the genes coding for the transcription factors MYB28 and MYB29 reportedly involved in controlling the AG biosynthetic pathway in response to biotic and abiotic stress (Hirai et al., 2007; Sønderby et al., 2010) (**Figures S5E, F**). These results are in agreement with those obtained by Ferrari et al. (2007) in a full-genome expression analysis of *Arabidopsis* plants treated with *B. cinerea*, where the genes encoding enzymes involved in the biosynthesis of Trp and indole compounds were up-regulated, whereas most genes encoding enzymes involved in the biosynthesis of AG, like *CYP79F1*, *REF2*, and *UGT74C1* (Hansen et al., 2001; Hemm et al., 2003; Gachon et al., 2005), were repressed or not significantly affected.

In conclusion, the results of this work show that an *Arabidopsis ugt80A2;B1* mutant is more resistant to the infection by the necrotrophic fungus *B. cinerea* than the corresponding WT plants. This effective response against *B. cinerea* seems to be mediated by the enhanced levels of some defense secondary metabolites, such as camalexin and probably also IG, in the *ugt80A2;B1* mutant compared to the WT. The biosynthesis of these compounds is regulated by a set of transcription factors that can be activated by the high levels of JA present in the mutant, which in turn would induce the expression of some defense genes, like *PDF1.2* and *PR4*. However, the upstream mechanisms that trigger this response, including the membrane localized signal transduction steps, remain elusive. Steryl glycosides are enriched in the plasma membrane lipid rafts or DRM, which control dynamic protein interactions in a specific sterol-lipid environment (Zauber et al., 2014). The biological function of these microdomains has been linked to signaling and transport, since proteomic analysis have identified several proteins involved in these processes in the DRM (Shahollari and Berghöfer, 2004; Kierszniowska et al., 2009). Thus, it might be hypothesized that an altered composition of glycosylated sterols in the membrane rafts might affect their structure and function, resulting in an indirect differential modulation of some signaling pathways, such as those described in this work. The identification of some immunity-related proteins whose levels are increased in the DRM of the *ugt80A2;B1* mutant (Zauber et al., 2014) would support this hypothesis. These proteins include PERK1, a membrane receptor-like kinase involved in the general perception and response to wounding and/or pathogen stimulus (Silva and Goring, 2002); PLC2 (phospholipase C2), a protein that plays a role in MAMP-triggered immunity by modulating ROS production (D’Ambrosio et al., 2017); and AtRBOHD, a protein required for ROS production induced by DAMPs and pathogen attack (Liu and He, 2016). An alternative possibility is that the resistance phenotype observed in the *ugt80A2;B1* mutant could be due to a defect in a signaling role mediated directly by SGs, as described for the pleiotropic developmental phenotypes observed in different sterol biosynthesis mutants (Schrick et al., 2000; Schrick et al., 2002; He et al., 2003). The dissection of the activated transduction pathways and the identification of their different components will provide further insights about the mechanism of action by which glycosylated sterols may modulate the plant defense response against pathogen attack.

## Data Availability Statement

All datasets generated for this study are included in the manuscript and the **Supplementary Files**.

## Author Contributions

TA, AF, VF, and AB conceived and designed the research; NC and VP performed the infection studies and metabolites analysis; NC and ÁC conducted the gene expression analyses. NC, VP, MA, VF, AF, and TA collected and analyzed data. TA and AF wrote the manuscript.

## Funding

This work was funded by grants AGL2017-88842-R from FEDER/Ministerio de Ciencia, Innovación y Universidades-Agencia Estatal de Investigación (Spain), 2017SGR710 from the Generalitat de Catalunya, and by the CERCA Programme of the Generalitat de Catalunya. We also acknowledge financial support from the Spanish Ministerio de Economía y Competitividad through the “Severo Ochoa Programme for Centres of Excellence in R&D” 2016-2019 (SEV-2015- 0533). Research in VF laboratory has been funded by grant UJI-B2016-43 from the University Jaume I programme.

## Conflict of Interest

The authors declare that the research was conducted in the absence of any commercial or financial relationships that could be construed as a potential conflict of interest.
