# Supplementary figures and images for: Inactivation of UDP-Glucose Sterol Glucosyltransferases Enhances Arabidopsis Resistance to Botrytis cinerea
Source: Front Plant Sci. 2019 Sep 27;10:1162. doi: 10.3389/fpls.2019.01162 (PMC6776639; doi:10.3389/fpls.2019.01162)

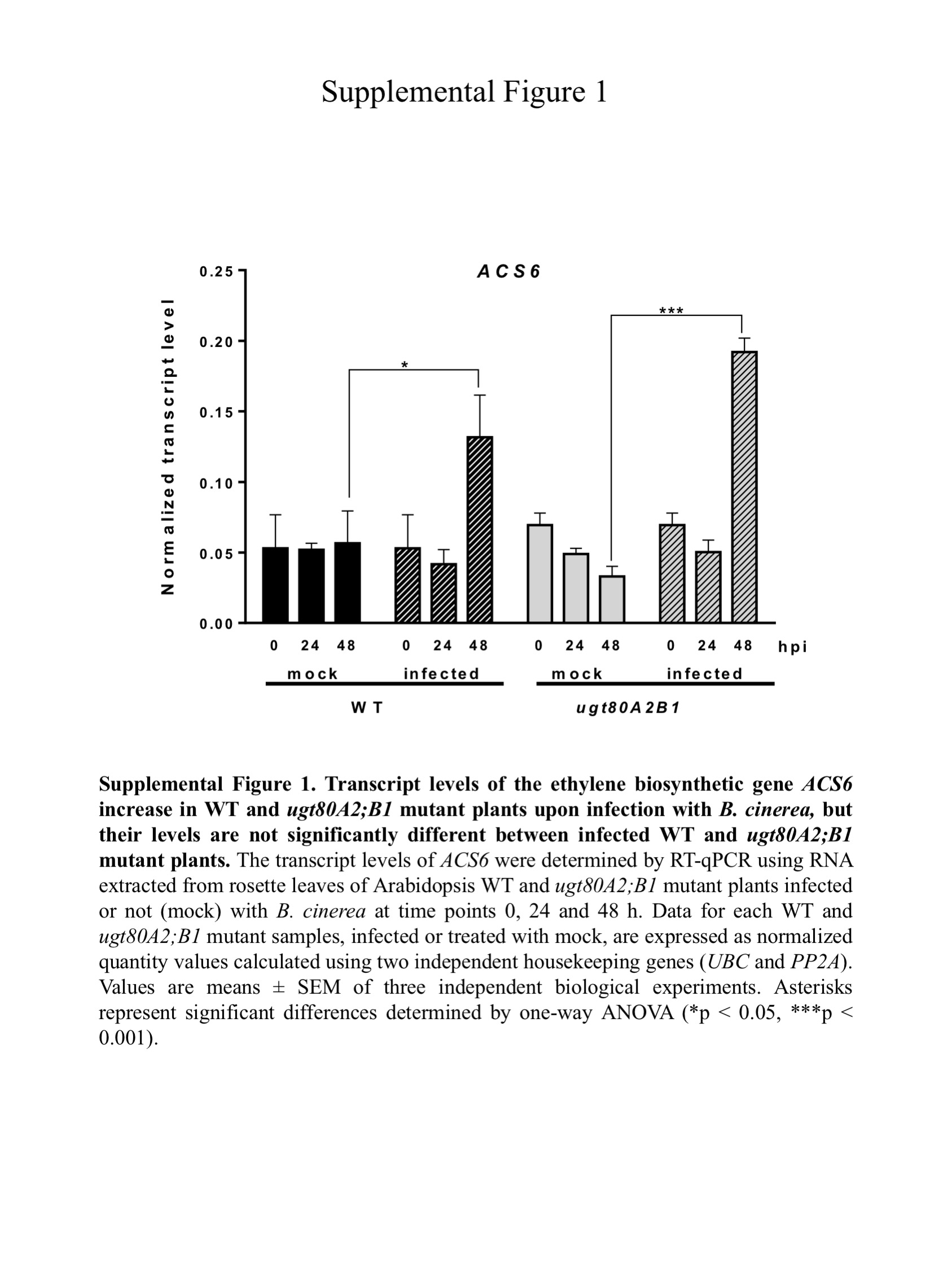

Supplement: Supplementary file 1 [file Image_1.jpeg]

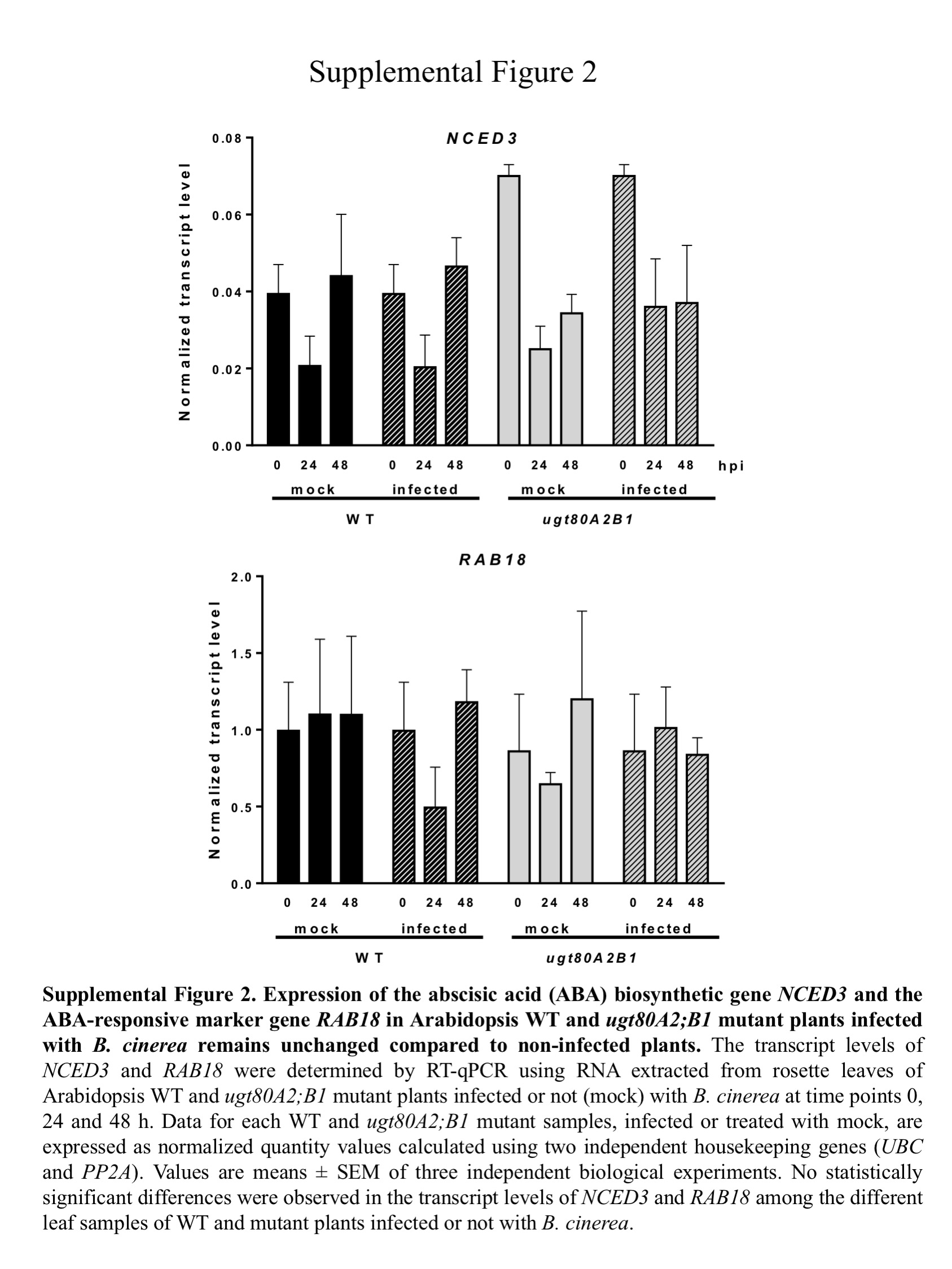

Supplement: Supplementary file 2 [file Image_2.jpeg]

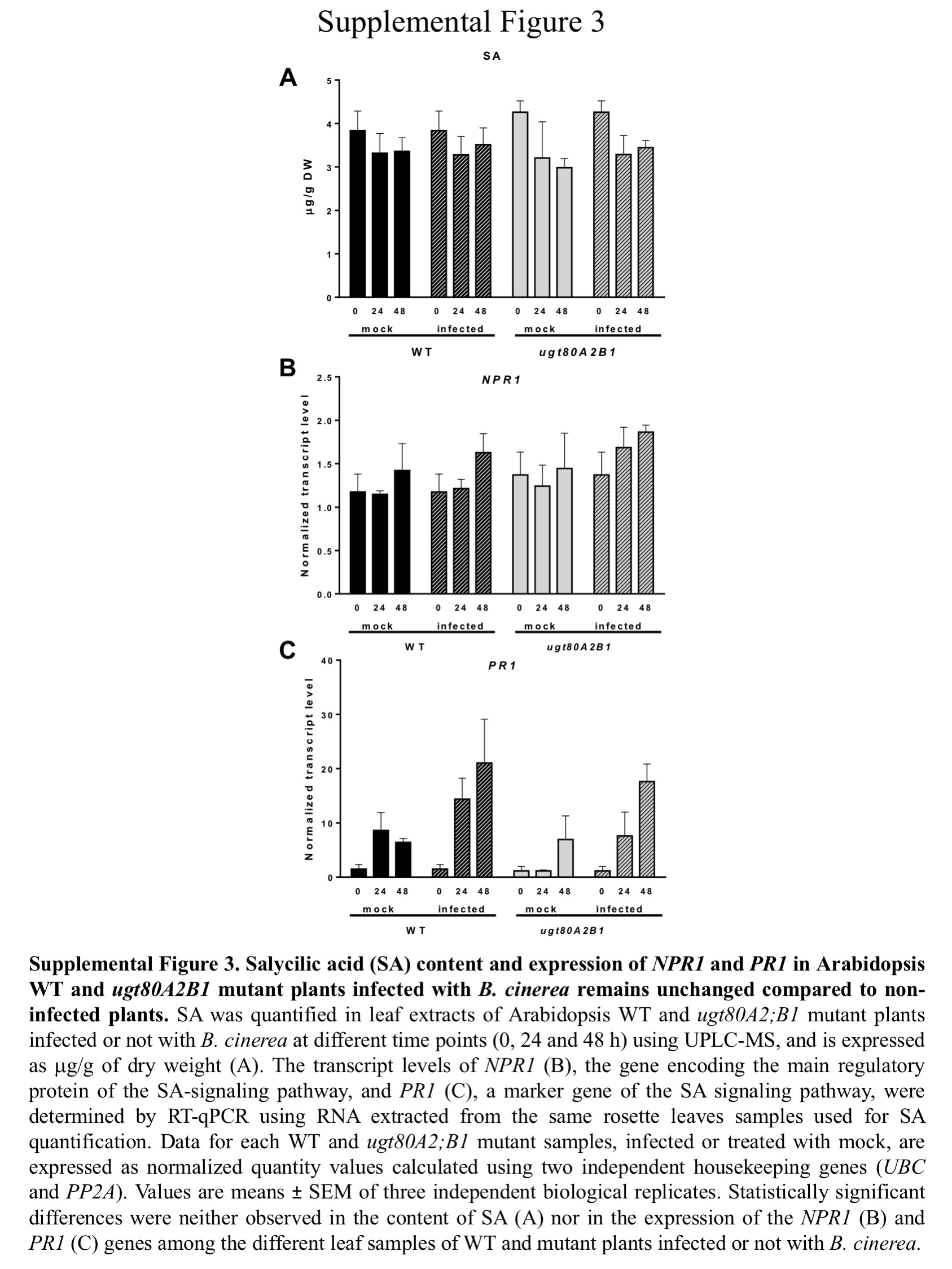

Supplement: Supplementary file 3 [file Image_3.jpeg]

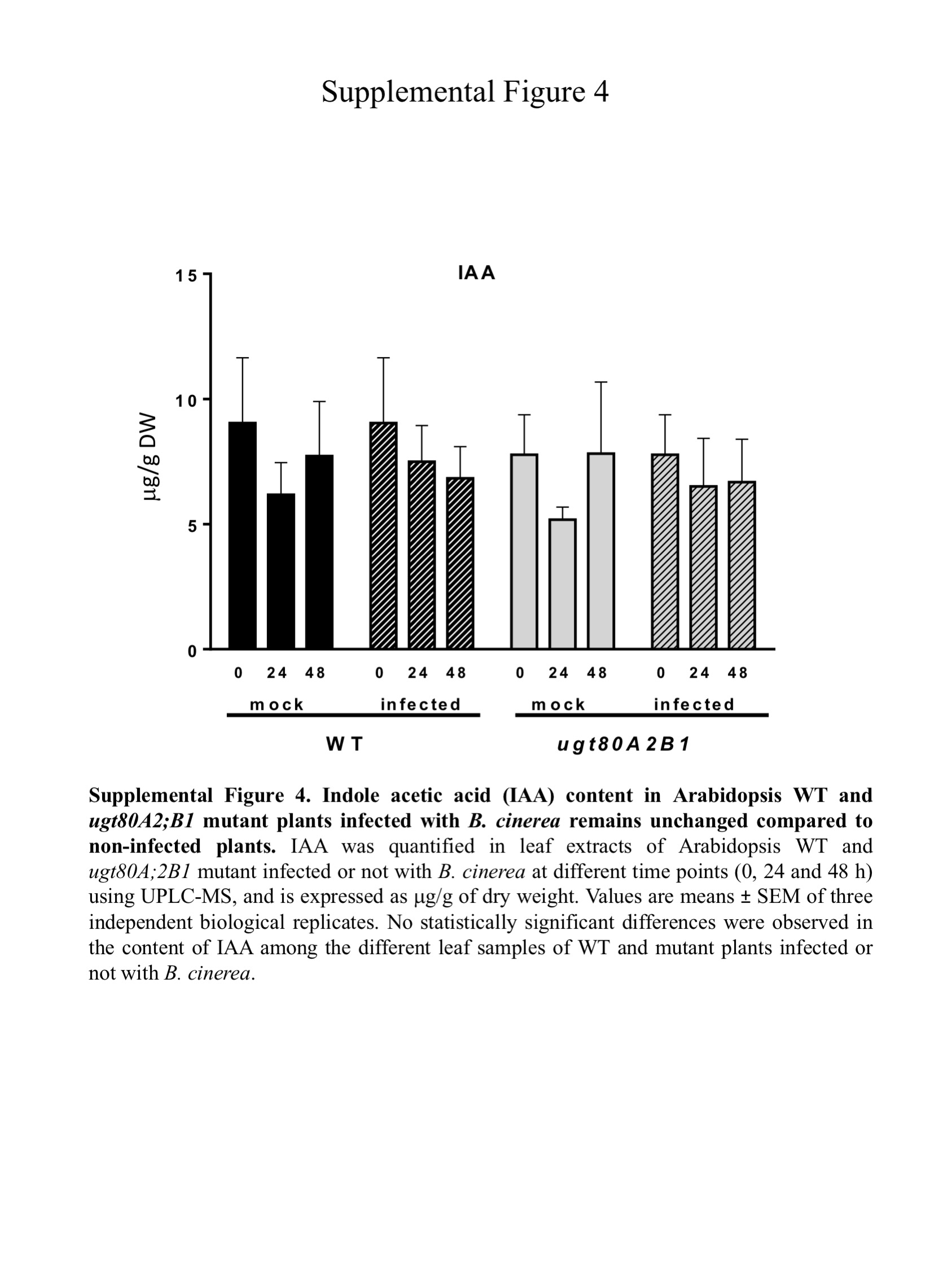

Supplement: Supplementary file 4 [file Image_4.jpeg]

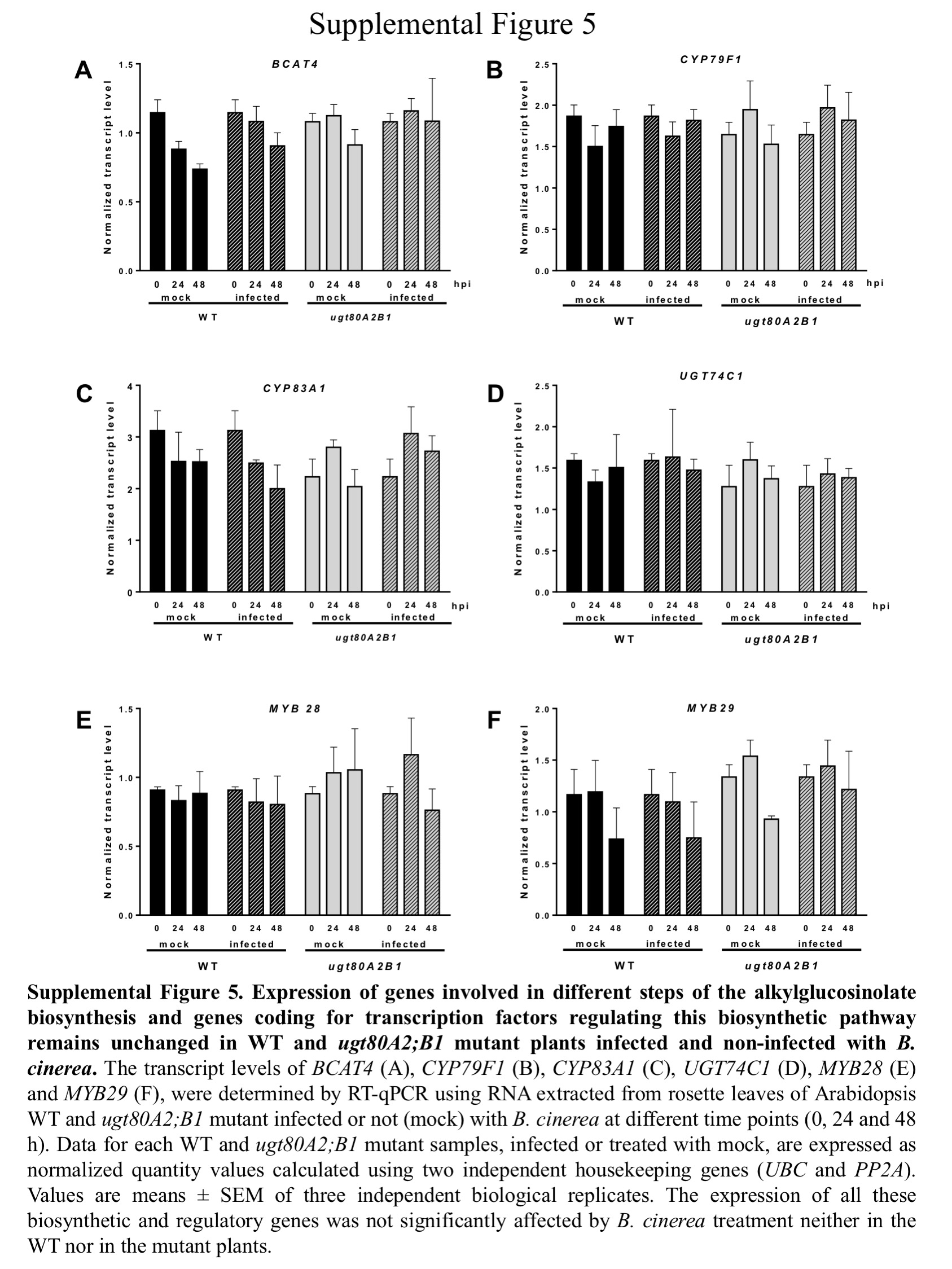

Supplement: Supplementary file 5 [file Image_5.jpeg]
