# Supplementary material for: Inactivation of UDP-Glucose Sterol Glucosyltransferases Enhances Arabidopsis Resistance to Botrytis cinerea
Source: Front Plant Sci. 2019 Sep 27;10:1162. doi: 10.3389/fpls.2019.01162 (PMC6776639; doi:10.3389/fpls.2019.01162)
Supplement: Supplementary file 6 [file Table_1.docx]

**Supplemental Table S1.** List of primers used in high-throughput quantitative PCR gene expression analysis.

| Gene | Accession number | primer | Sequence |
| --- | --- | --- | --- |
| ACS6 | At4g11280 | forward | GTTCCAACCCCTTATTATCC |
|  |  | reverse | CCGTAATCTTGAACCCATTA |
| ANAC042 | At2g43000 | forward | CACGAAAACCGACTCTCC |
|  |  | reverse | GTTTGGTGGTAAGATGGTTG |
| BCAT4 | At3g19710 | forward | CTGTACTGGCACTGCTTCCA |
|  |  | reverse | ATAGCTTCGCAGCCAATGTT |
| CYP71A13 | At2g30770 | forward | GGGTAGAGGCTGGACCAAAT |
|  |  | reverse | ACAACCGAAGATGGAAATGC |
| CYP71B15/PAD3 | At3g26830 | forward | GGTACGGGATAAATCTCTATGA |
|  |  | reverse | AGATACAGTCGATGAACCTAC |
| CYP79B2 | At4g39950 | forward | AACAAAAAGAAACCGTATCTGCCAC |
|  |  | reverse | TCCTAACTTCACGCATGCTATCTC |
| CYP79F1 | At1g16410 | forward | CTTGGTCACACCAGACGAAA |
|  |  | reverse | CCATGTTATTTGCCGGATTA |
| CYP81F2 | At5g57220 | forward | GTCACAGGGAGACGCTACTAC |
|  |  | reverse | CACCACTGTTGTCATTGATGTC |
| CYP83A1 | At4g13770 | forward | AGTCAAGCCCGAAACCGAGAG |
|  |  | reverse | GCAGTATCTGTTCCCGCCACTA |
| CYP83B1 | At4g31500 | forward | GGCAACAAACCATGTCGTATCAAG |
|  |  | reverse | CGTTGACACTCTTCTTCTCTAACCG |
| MYB28 | At5g61420 | forward | TCCCTGACAAATACTCTTGCTGAAT |
|  |  | reverse | CATTGTGGTTATCTCCTCCGAATT |
| MYB29 | At5g07690 | forward | CACGCATCTCAAAAAGCTCCTG |
|  |  | reverse | CTCCGGTACATTTTTGGAGCTTG |
| MYB51 | At1g18570 | forward | CTACAAGTGTTTCCGTTGACTCTGAA |
|  |  | reverse | ACGAAATTATCGCAGTACATTAGAGGA |
| NCED3 | At3g14440 | forward | CAGCTTGTAGCTTTTGGGCTGTA |
|  |  | reverse | TAACAGAAACCAGCTGAGCTCGA |
| NPR1 | At1g64280 | forward | CCGGAAGAGCTTGTTAAAGAGA |
|  |  | reverse | ATCCGAGTCAAGTGCCTTATGT |
| PDF1.2 | At5g44420 | forward | CGAGAAGCCAAGTGGGACAT |
|  |  | reverse | TCCATGTTTGGCTCCTTCAA |
| PP2AA3 | At1g13320 | forward | GCGGTTGTGGAGAACATGATACG |
|  |  | reverse | GAACCAAACACAATTCGTTGCTG |
| PR1 | At2g14610 | forward | TTCTTCCCTCGAAAGCTCAA |
|  |  | reverse | AAGGCCCACCAGAGTGTATG |
| PR4 | At3g04720 | forward | TGTGAGAATAGTGGACCAATGC |
|  |  | reverse | CCATCGGTGTCTATTTGATTGA |
| RAB18 | At5g66400 | forward | CGGGACTGAAGGCTTTGGA |
|  |  | reverse | CACCACTTTCCTTGTGGAGTTG |
| UBC21 | At5g25760 | forward | TCAAATGGACCGCTCTTATC |
|  |  | reverse | CACAGACTGAAGCGTCCAAG |
| UGT74B1 | At1g24100 | forward | ATCCTGAGCATGGCAGAGTT |
|  |  | reverse | CCCTTCAAAGCCATTAACGA |
| UGT74C1 | At2g31790 | forward | CCTGACCGATTTCATCTCTAGTGC |
|  |  | reverse | TGGCTATGTCCAATGCAAAGGG |
| UGT80A2 | At3g07020 | forward | CACCAATCCCAGTGGATGAA |
|  |  | reverse | TGTCTCTGCACTGCTCTTTAC |
| UGT80B1 | At1g43620 | forward | ACAACGATCGTGCCATTCT |
|  |  | reverse | CTGAGCTGAGCTATCGGTATTG |
| VSP2 | At5g24770 | forward | TCAGTGACCGTTGGAAGTTGT |
|  |  | reverse | GTTCGAACCATTAGGCTTCAATAT |
| WRKY33 | At2g38470 | forward | GTGATATTGACATTCTTGACGA |
|  |  | reverse | GATGGTTGTGCACTTGTAGTA |
